# Cytoglobin Attenuates Neuroinflammation in Lipopolysaccharide-Activated Primary Preoptic Area Cells *via* NF-κB Pathway Inhibition

**DOI:** 10.3389/fnmol.2019.00307

**Published:** 2019-12-12

**Authors:** Bruna R. B. Gomes, Gabriela Luna S. de Sousa, Daniela Ott, Jolanta Murgott, Marcelo V. de Sousa, Paulo E. N. de Souza, Joachim Roth, Fabiane H. Veiga-Souza

**Affiliations:** ^1^Laboratory of Protein Chemistry and Biochemistry, Department of Cell Biology, Institute of Biology, University of Brasília, Brasília, Brazil; ^2^School of Ceilandia, University of Brasília, Brasília, Brazil; ^3^Veterinary Physiology, Faculty of Veterinary Medicine, Justus-Liebig-University of Giessen, Giessen, Germany; ^4^Laboratory of Electron Paramagnetic Resonance, Institute of Physics, University of Brasília, Brasília, Brazil

**Keywords:** cytoglobin, neuroinflammation, fever, preoptic area, primary culture, hypothalamus

## Abstract

Cytoglobin (Cygb) is a hexacoordinate protein, associated with the transport of oxygen, nitric oxide scavenging, tumor suppression and protection against oxidative stress and inflammation. This protein is expressed in brain areas including the preoptic area (POA) of the anterior hypothalamus, the region responsible for the regulation of body temperature. In this study, we show that Cygb is upregulated in the rat hypothalamus 2.5 h and 5 h after intravenous administration of lipopolysaccharide (LPS). We investigated the effect of treatment with Cygb in POA primary cultures stimulated with LPS for 4 h. The levels of tumor necrosis factor-alpha (TNF-α) and interleukin-6 (IL-6) were measured and the results showed that Cygb reduced the concentrations of both cytokines. We further observed a decrease in immunoreactivity of the inflammatory transcription factor nuclear factor-κB (NF-κB), but not NF-IL6 and STAT3, in the nucleus of Cygb-treated POA cells. These findings suggest that Cygb attenuates the secretion of IL-6 and TNF-α in LPS-stimulated POA primary cultures *via* inhibition of the NF-κB signaling pathway, indicating that this protein might play an important role in the control of neuroinflammation and fever.

## Introduction

Systemic administration of lipopolysaccharide (LPS) induces a number of brain-controlled sickness responses, such as anorexia, adipsia, reduced locomotor activity, and fever (Dantzer et al., 1998; Damm et al., 2013). LPS binds to the Toll-like receptor (TLR) member TLR4 in cells of both the peripheral and central nervous system, triggering intracellular cascades of events. These include activation of pro-inflammatory transcription factors, and production and release of several mediators, such as cytokines, chemokines, and prostaglandins (PGs), which are implicated in the aforementioned brain-controlled sickness responses (Damm et al., 2013; Roth and Blatteis, 2014; Zampronio et al., 2015).

Using high-resolution mass spectrometry-based quantitative proteomics, we recently demonstrated that during LPS and PG E_2_-induced fever an orchestrated response, involving changes of several proteins associated with inflammatory and metabolic pathways, occurs in the hypothalamus of rats (Firmino et al., 2018). Among the inflammatory pathway components activated, we detected mitogen-activated protein kinases, nuclear factor-κB (NF-κB), arachidonic acid, adenylate cyclase, and calcium signaling. In addition to the proteins related to these well-known inflammatory pathways, we have identified a distinct protein that is not directly associated with any of these classical pathways, but significantly increases after induction of fever by both LPS and by PGE_2_. This protein, named cytoglobin (Cygb), shows features that support its potential involvement in neuroinflammation and fever regulation.

Cygb is a hexacoordinate globin ubiquitously expressed in various organs and cell types (Burmester et al., 2000, 2002). In the brain, it has been found only in distinct areas, including the hypothalamic preoptic area (POA; Hundahl et al., 2010), which is known to be the major thermoregulatory center in mammals (Roth and Blatteis, 2014; Zampronio et al., 2015). At a subcellular level, Cygb is expressed in the nucleus, mitochondria, cytoplasm and neuronal dendrites and axons (Hundahl et al., 2010; Oleksiewicz et al., 2011).

Cygb has been implicated in oxygen transport, nitric oxide scavenging, tumor suppression and protection against oxidative stress and inflammation (Ou et al., 2018; Yassin et al., 2018). Consistent with its described functions, the expression of Cygb is upregulated in conditions associated with hypoxia, oxidative stress and inflammation (Guo et al., 2007; Tae et al., 2017; Yassin et al., 2018).

Indeed, in a study of an alcoholic liver disease model, treatment with recombinant human Cygb significantly inhibited Kupffer cell (KC) proliferation and tumor necrosis factor-alpha (TNF-α) expression, in LPS-stimulated KCs. Cygb also inhibited LPS-induced NADPH oxidase activity and ROS, NO, and O_2_^•-^ generation (Wen et al., 2017). In addition, Cygb-deficient mice exhibited increased inflammation (Thuy et al., 2015; Van Thuy et al., 2017), as well as increased expression of TNF-α mRNA and many genes associated with inflammation (Yassin et al., 2018). These findings suggest that Cygb has a protective role in inflammation. However, its effects on neuroinflammation, especially in the cells of the POA, have not been investigated. Therefore, the purpose of the present study was to validate the increase in hypothalamic Cygb expression after a pyrogenic dose of LPS, and to investigate its function by examining levels of inflammatory cytokines (TNF-α and interleukin-6, IL-6) and the activation of transcription factors such as NF-IL6, STAT3, and NF-κB, in LPS-activated POA cells co-treated with recombinant rat Cygb.

## Materials and Methods

### Animals

Fever experiments were conducted in male and female Wistar rats (180–200 g). The study was carried out in accordance with the guidelines of the National Council for the Control of Animal Experimentation (CONCEA) and the Guide for the Care and Use of Laboratory Animals of the Institute for Laboratory Animal Research (2011). The protocol was approved by the Animals Research Ethics Committee of the University of Brasília (approval number 60/2018). Animals were housed at 24 ± 1°C, under 12:12 h light-dark cycle (lights on at 7 am), with free access to food chow and tap water.

For the preparation of primary cultures from the POA, Wistar rat pups (4–6 d old) of both sexes were obtained from an in-house breeding colony and used for all experiments, with parent animals originating from Charles River WIGA (Sulzfeld, Germany). Animal care, breeding, and experimental procedures were conducted according to the guidelines approved by the Hessian Ethics Committee (approval numbers GI 468_M and GI 487_M). Room temperature was maintained at 22 ± 1°C with a relative humidity of 50%. Artificial lights were on from 7 am to 7 pm.

### Core Temperature Measurement

Body core temperature was measured in conscious adult rats using data loggers (Subcue, Calgary, AB, Canada) as previously described (Gomes et al., 2018). To induce fever, rats received an intravenous injection of LPS at a dose of 5 μg/kg, determined according to previous studies showing both a moderate febrile response induction (Gomes et al., 2018) and Cygb increase in the hypothalamus (Firmino et al., 2018). The animals were harvested by decapitation 2.5 h or 5 h after the LPS injection, and the hypothalami were dissected from the brain and immediately frozen at −80°C until further processing.

### Western Blot Analysis

Freshly thawed hypothalamus samples were prepared as previously described (Firmino et al., 2018). Western blots for Cygb were performed using 12% sodium dodecyl sulfate-polyacrylamide gel electrophoresis (SDS-PAGE). Thirty micogram of protein was loaded per well. We used the primary Cygb antibody raised in rabbit (1:1,000, Boster Biological Technology, Pleasanton, CA, USA). Quantitative analysis was performed using ImageJ software (National Institute of Health). Data were normalized to β-actin levels.

### Preoptic Area Primary Cell Cultures

Primary rat cultures were prepared as previously described (Soares et al., 2013). To stimulate the cells, they were incubated with LPS (Sigma–Aldrich, St. Louis, MO, USA; 10 μg/ml) or recombinant rat Cygb (Cusabio Technology, Houston, TX, USA; 10 and 20 μg/ml) for 4 h. The concentrations of LPS and Cygb used were based on previous studies (Wen et al., 2017; Leisengang et al., 2018). PBS was used as a negative control. The supernatant was harvested and stored at −45°C for later measurement of cytokines levels. Cells were used for immunocytochemistry.

### Measurements of TNF-α and IL-6

Cytokines were measured in the supernatant of POA primary cell cultures. TNF-α and IL-6 levels were determined by bioassay, based on the cytotoxic effect of TNF-α on mouse fibrosarcoma cell line WEHI 164 subclone 13, and the dose-dependent growth stimulation of IL-6 on the B9 hybridoma cell line, as previously reported (Ott et al., 2010; Soares et al., 2013; Simm et al., 2016). The detection limits of the assays, after considering the dilution of samples, proved to be 6.0 pg/ml for TNF-α and 3.0 IU/ml for IL-6 (Ott et al., 2010).

### Cell Viability

Viability of primary POA cells after treatment with LPS and Cygb was determined using the trypan blue exclusion assay, as described by Perry et al. (1997).

### Immunocytochemistry

Immunocytochemistry was performed in primary POA cells exposed to LPS for 4 h with or without Cygb. Nuclear translocation of the transcription factors NF-IL6 and NF-κB in microglial cells and STAT3 in astrocytes was examined using previously published protocols (Leisengang et al., 2018). Primary antibodies were used in the following concentrations: anti-NF-IL6 (1:4,000), anti-NF-κB (1:2,000), anti-STAT3 (1:4,000), anti-GFAP (1:1,000), anti-ED-1 (1:1,000). For visualization of nuclei, DAPI was used (1:1,000). The cells were incubated with fluorophore-coupled secondary antisera (Alexa Fluor 488 donkey anti-mouse IgG, 1:500 and Cy3-conjugated donkey anti-rabbit IgG, 1:2,000).

Cells were examined and photographed using a Nikon TMD epifluorescent microscope equipped with the appropriate filter sets. The intensity of one of the three color channels, which represented nuclei NF-κB, NF-IL6, and STAT3 signals, was quantified using MetaMorph microscopic imaging software (Molecular Devices, San Jose, CA, USA).

### Statistics Analyses

All values are expressed as mean ± SEM. Statistical analyses was performed using ANOVA, followed by Bonferroni’s multiple comparisons test. Differences were considered significant at a value of *p* < 0.05. The reproducibility of the data was confirmed by at least three independent experiments.

## Results

### Cytoglobin Upregulation in Rat Hypothalamus After Injection of a Pyrogenic LPS-Dose

**Figure 1:**
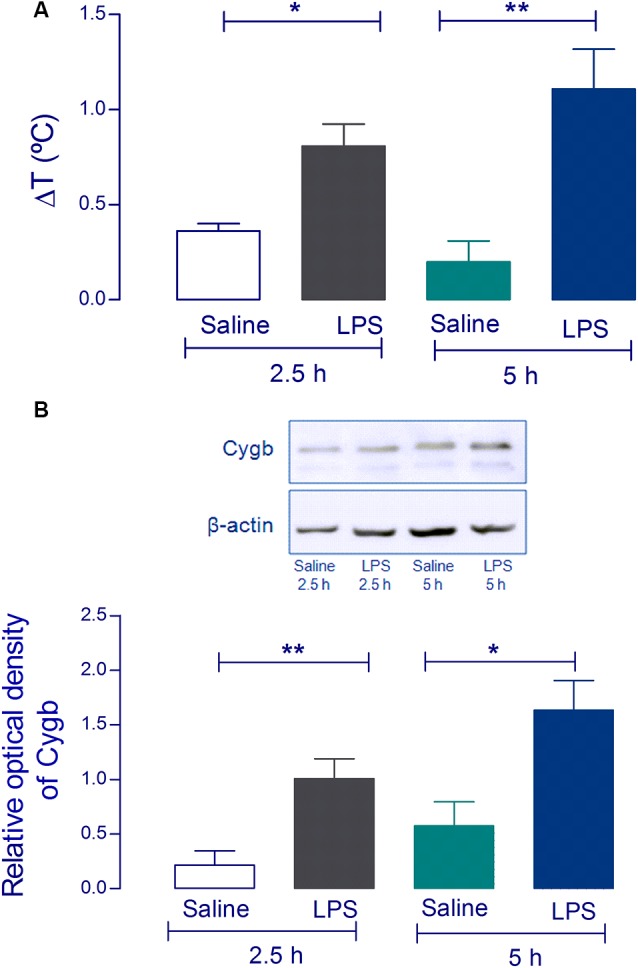
Cytoglobin (Cygb) expression is increased in rat hypothalamus after intravenous injection of lipopolysaccharide (LPS). Rat hypothalamus tissue was collected 2.5 h and 5 h after the intravenous LPS injection (5 μg/kg). The bars represent the means ± SEM of the change in body temperature (ΔT, in °C), with respect to the basal temperature at the moment of euthanasia of the animals **(A**; *n* = 4). *p < 0.05 or **p < 0.01 compared with the saline groups. Protein levels of Cygb at the hypothalamus collected 2.5 h and 5 h were analyzed by Western blotting, showing increased amounts of Cygb in both times tested **(B)**. β-actin was used as the loading control. The bars represent mean ± SEM of four animals per group. *p < 0.05 or **p < 0.01 when compared to the corresponding value of the saline group.

Using Western blot analysis, we first attempted to validate the increase of Cygb in the hypothalamus of animals challenged with a pyrogenic dose (5 μg/kg) of intravenous LPS. The hypothalami were collected 2.5 and 5 h after injection when LPS had induced significant increases in core temperatures (Figure 1A). Consistent with our previous proteomic results (Firmino et al., 2018) we detected significant increases in Cygb in animals challenged with LPS, at both times tested (Figure 1B).

### Cytoglobin Attenuates the Secretion of Cytokines Induced by LPS

**Figure 2:**
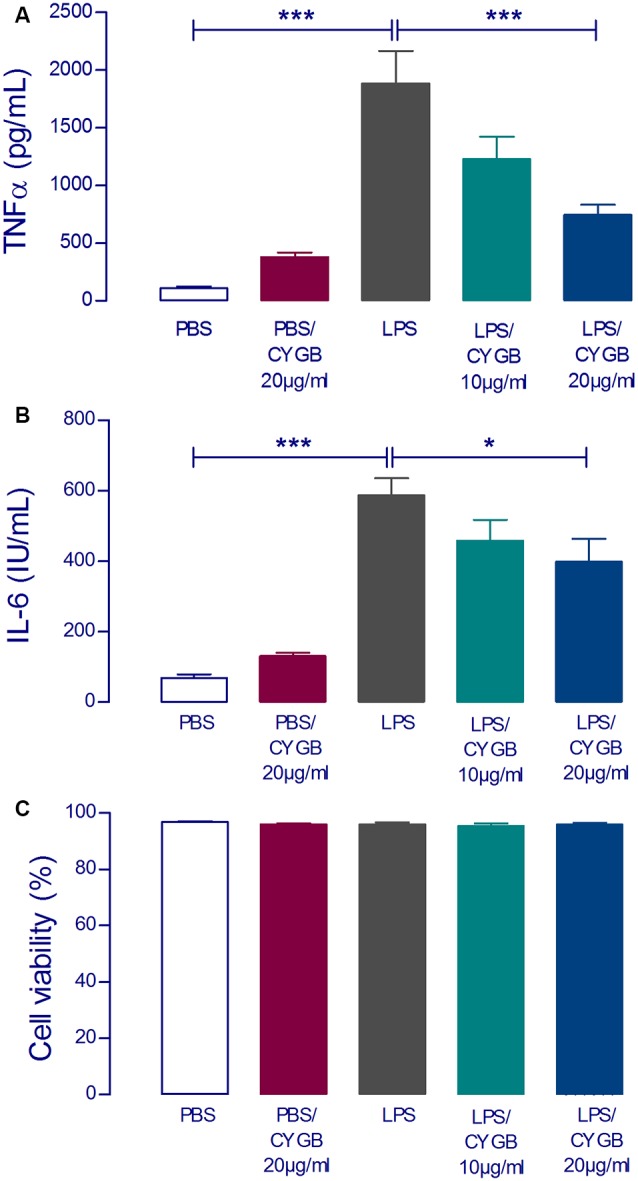
LPS-induced tumor necrosis factor-alpha (TNF-α) and interleukin-6 (IL-6) concentrations in supernatants of rat preoptic area (POA) primary cultures under the influence of Cygb. POA primary cultures cultured on poly-L-lysine-coated glass coverslips, were incubated for 240 min with fresh medium containing PBS (negative control), LPS at the concentration of 10 μg/ml (positive control) or LPS (10 μg/ml) plus Cygb (10 μg/ml or 20 μg/ml). LPS caused a significant increase in TNF-α and IL-6 concentrations in the supernatants of POA primary cultures and the co-treatment with Cygb prevent significantly this increase at the dose 20 μg/ml for TNF-α **(A)** and IL-6 **(B)**. The viability of the cells is not altered in any tested group **(C)**. Columns (means of 3–4 samples from three to six independent experiments) represent means with SEM (significant difference vs. LPS control group; *p < 0.05; ***p < 0.001).

To examine the effect of Cygb on LPS-induced neuroinflammatory responses in POA cells, levels of the inflammatory cytokines TNF-α and IL-6 were measured (Figure 2). The secretion of both cytokines was significantly increased in LPS (10 μg/ml) stimulated POA cells compared with the control group. This effect of LPS was attenuated by co-treatment of cells with Cygb (20 μg/ml). The inhibitory effects on the secretion of IL-6 and TNF-α were not due to a reduction in cell viability since incubation with Cygb did not change this parameter, compared to the control group (Figure 2C).

### Cytoglobin Regulates the Activation of NF-κB After LPS Treatment

**Figure 3:**
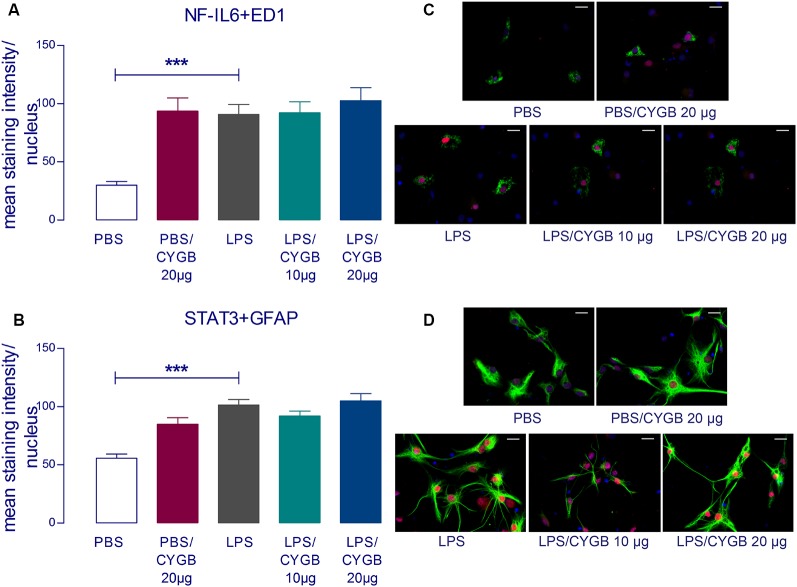
Cygb does not affect the nuclear NF-IL6 and STAT3 immunoreactivity in microglia and astrocytes, respectively. Immunocytochemistry was proceeded in coverslips using the NF-IL6 antiserum in ED1-positive microglia **(A,C)** and STAT3 antiserum in GFAP-positive astrocytes **(B,D)**. The immunoreactivity was enhanced in the cells treated with LPS (10 μg/ml) compared to the PBS group and the co-treatment with Cygb (10 μg/ml or 20 μg/ml) does not affect this. In panel **(C)**, triple labeling for ED-1 (in green), NF-IL-6 (in red) and cellular nuclei by DAPI (in blue) allowed localization of NF-IL6 immunoreactivity in the nuclei of microglial cells. In panel **(D)**, triple labeling for GFAP (in green), STAT3 (in red) and cellular nuclei by DAPI (in blue) allowed localization of STAT3 immunoreactivity in the nuclei of astrocytes. Secondary antisera employed were coupled to fluorophores Alexa-488 (green label) and Cy3 (red label). Scale bars: 20 μm. The average intensities of the signals within the active region of interest (here: cell nuclei) were expressed as gray values. Columns represent means of the intensities measured in treated cultures derived from two independent preparations (***p < 0.001).

**Figure 4:**
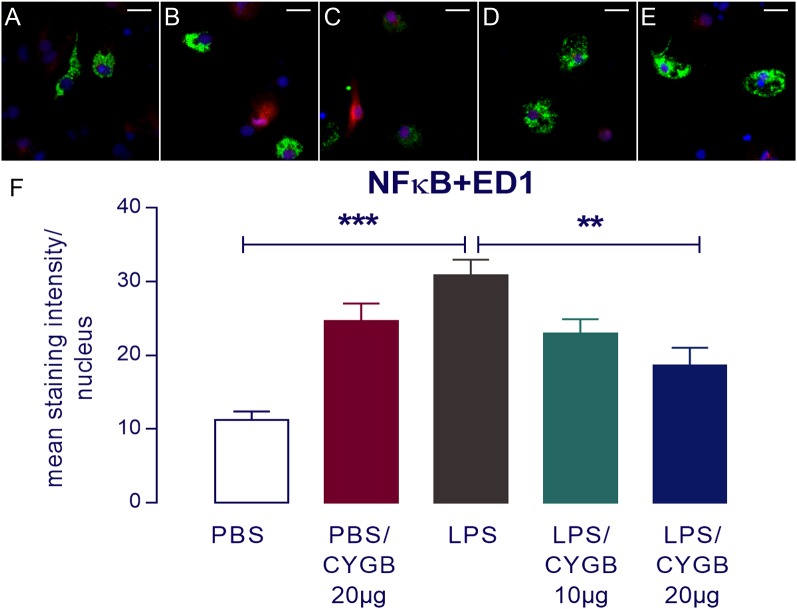
Nuclear factor-κB (NF-κB) immunoreactivity in microglia in the primary microculture of rat POA in the response of stimulation of LPS and treatment with Cygb. NF-κB immunoreactivity was detected in ED1-positive microglia after 240 min stimulation of primary cultures with medium containing 10 μg/ml LPS **(C)**, while the immunoreactivity in the PBS **(A)** groups was discrete. Triple labeling for ED-1 (in green), NF-κB (in red) and cellular nuclei by DAPI (in blue) demonstrate localization of NF-κB mainly to the nucleus of LPS-treated ED1-positive cells **(C)**, while there was a decrease NF-κB immunoreactivity in the cells treated with Cygb (20 μg/ml; **E)**. There are no significant changes in immunoreactivity in PBS + Cygb 20 μg or LPS + Cygb 10 μg groups (**B,D**, respectively). Secondary antisera employed were coupled to fluorophores Alexa-488 (green label) and Cy3 (red label). Scale bars: 20 μm. The average intensities of the signals within the active region of interest (here: cell nuclei) were expressed as gray values **(F)**. Columns represent means of the intensities measured in treated cultures derived from two independent preparations (**p < 0.01 or ***p < 0.001).

LPS-induced cytokine secretion by hypothalamic cells occurs *via* activation of inflammatory transcription factors (reviewed by Rummel, 2016). As expected, POA cells stimulated with LPS for 4 h showed increased immunoreactivity for NF-IL6, STAT3, and NF-κB, when compared to the PBS group (Figures 3, 4). As Cygb reduced TNF-α and IL-6 secretion, we investigated whether these inhibitory effects were due to a change in the activation of transcription factors. We found that co-treatment of POA cells with LPS and Cygb did not alter immunoreactivity for NF-IL6 and STAT3, but significantly decreased the intensity of NF-κB signals in microglial cells (Figure 4). This result suggests that Cygb exerts an anti-neuroinflammatory effect by inhibiting the NF-κB signaling pathway.

Surprisingly, we also observed increased immunoreactivity for all transcription factors in the Cygb + PBS group when compared to PBS alone (Figures 3, 4). In line with this observation, there was a strong tendency for higher cytokine concentrations in the supernatants of cells treated with Cygb +PBS, when compared to PBS alone. Still, this effect was not significant (Figure 2) and the inhibitory effect of Cygb on LPS-induced release of cytokines was manifest in each of the independent experiments. Since the recombinant rat Cygb used in the present study was expressed in *E. coli*, we cannot exclude the possibility that some residual level of endotoxin was still present and caused the effect of Cygb on nuclear immunoreactivity of transcription factors. Despite this, our results show undoubtedly that Cygb was able to reduce NF-κB signal intensity induced by LPS.

## Discussion

In this study, we demonstrated the anti-neuroinflammatory effect of Cygb in LPS-activated POA-cells by attenuation of pro-inflammatory cytokine secretion by inhibition of the NF-κB pathway. In addition, we confirmed previous proteomic results by showing up-regulation of Cygb after animals were challenged with LPS (Firmino et al., 2018).

Cygb belongs to a family of globins (Burmester et al., 2000, 2002) and is expressed in the POA of the hypothalamus (Hundahl et al., 2010), which is known to be the major thermoregulatory center in mammals (Roth and Blatteis, 2014; Zampronio et al., 2015). Our results from Western blot analyses showed that Cygb is increased at 2.5 h and 5 h after intravenous administration of LPS. These times are compatible with the characteristic peaks of LPS-induced fever (Romanovsky et al., 1998). These findings validate our mass spectrometry-based proteomics data that showed upregulation of Cygb in the hypothalamus after animals were challenged with LPS and PGE_2_ (Firmino et al., 2018). Taken together, these data compelled us to explore the role of Cygb in the regulation of neuroinflammation, using LPS-stimulated POA primary cultures.

Cygb was first identified in the 2000s, as a ubiquitously protein with a potential protective function during conditions of nitro-oxidative stress (Burmester et al., 2002; Trent and Hargrove, 2002). Accumulating recent evidence suggests a complex interplay between nitro-oxidative stress and inflammation (Varga et al., 2015; Milatovic et al., 2017), and the study of endogenous antioxidants, such as Cygb, is important for understanding endogenous anti-inflammatory pathways. Systemic exposure to insults, such as toxins or bacterial infection, triggers an inflammatory response associated with the release of inflammatory mediators, such as free radicals, cytokines, and chemokines (Milatovic et al., 2017). As a NO scavenger, Cygb has been reported as an anti-inflammatory and antitumor agent (De Backer et al., 2018; Yassin et al., 2018). Ou et al. (2018) showed that Cygb prevents atherosclerosis by functioning as an NO dioxygenase enzyme and ROS scavenger, favoring the protection of the body against unbalanced homeostasis.

In the present study, we identified a decrease in levels of TNF-α and IL-6 in the supernatant of POA primary cultures after treatment with Cygb, revealing that this protein can regulate the neuroinflammation induced by LPS. Using a model of colitis in mice, Yassin et al. (2018) observed that Cygb-deficient mice developed more severe inflammation and that TNF-α increases Cygb mRNA expression in colonic epithelial cells, which might support a role for Cygb as a cytoprotective protein during inflammation. In addition, Cygb-deficient mice show elevated TNF-α and IL-6 in tumor and non-tumor tissue of the liver and lung (Thuy et al., 2011). These findings indicate that Cygb deficiency can trigger inflammation, which may contribute to increased susceptibility to cancer development (Roh et al., 2003; Park et al., 2010; Tsukamoto et al., 2018).

Our results indicate that Cygb did not change the nuclear immunoreactivity profile of the transcription factors STAT3 and NF-IL6 induced by LPS. However, we detected a significant reduction in immunoreactivity for NF-κB in cultures treated with Cygb. NF-κB is a transcription factor activated by TLR agonists and cytokines, such as TNF-α, and is thought to be a key regulator of neuroinflammation (Mincheva-Tasheva and Soler, 2013) and fever, since NF-κB-deficient mouse do not develop a fever after LPS injection (Kozak et al., 2006; Rummel, 2016). Accordingly, substances that can inhibit NF-κB activation are target candidates for the development of therapies to treat neuroinflammation and fever.

One limitation of the current investigation is that in the POA primary cell culture model, there is a considerable loss of endothelial cells during cell preparation, making it impossible to analyze the effect of Cygb on the levels of PGE_2_, an essential mediator of fever, since it is mainly produced by endothelial cells (Eskilsson et al., 2017). An additional limitation is that the amount of samples produced by the POA primary cells is insufficient to validate changes in cytokines and NF-κB by other methods such as Western blot.

In summary, we confirm that in the experimental model of fever induced by LPS, hypothalamic Cygb protein levels are increased. To investigate the functional role of Cygb, we used an *in vitro* model of primary POA cell cultures. Of note, Cygb attenuates the secretion of IL-6 and TNF-α, *via* inhibition of the NF-κB signaling pathway. In future studies, we intend to measure the levels of PGE_2_, as well as to validate the regulatory effect of Cygb on cytokines and transcription factors in animals treated with Cygb. In addition, it would be of interest to determine the interplay between antioxidative and anti-inflammatory actions of Cygb, to further examine its potential as a therapeutic target for the control of neuroinflammation and fever.

## Data Availability Statement

The datasets generated for this study are available on request to the corresponding author.

## Ethics Statement

The animal study was reviewed and approved by Animals Research Ethics Committee of the University of Brasília (approval number 60/2018).

## Author Contributions

FV-S conceived the project. BG, DO, JR and FV-S conceived and designed the experiments. BG, GS, DO, JM and FV-S performed the experiments. BG, DO, JM, JR and FV-S analyzed the data. BG and FV-S wrote the manuscript. MS, PS, DO and JR edited the manuscript. MS and PS contributed with advice and discussion. All authors approved the final version of the manuscript.

## Conflict of Interest

The authors declare that the research was conducted in the absence of any commercial or financial relationships that could be construed as a potential conflict of interest.
